# Evaluation of Antibacterial, Antineoplastic, and Immunomodulatory Activity of* Paullinia cupana* Seeds Crude Extract and Ethyl-Acetate Fraction

**DOI:** 10.1155/2016/1203274

**Published:** 2016-12-07

**Authors:** Lidiane Vasconcelos do Nascimento Carvalho, Marina Ferraz Cordeiro, Thiago Ubiratan Lins e Lins, Maria Clara Pinheiro Duarte Sampaio, Gabriela Souto Vieira de Mello, Valécia de Cassia Mendonça da Costa, Leila Larisa Medeiros Marques, Traudi Klein, João Carlos Palazzo de Mello, Isabella Macário Ferro Cavalcanti, Ivan da Rocha Pitta, Maira Galdino da Rocha Pitta, Moacyr Jesus Barreto de Melo Rêgo

**Affiliations:** ^1^Laboratory of Immunomodulation and New Therapeutical Approaches, Research Centre for Therapeutic Innovation Suely Galdino (NUPIT-SG), Federal University of Pernambuco, Recife, PE, Brazil; ^2^Pharmacy Department, Maringa State University, Maringa, PR, Brazil; ^3^Pharmacy Department, Ponta Grossa State University, Ponta Grossa, PR, Brazil; ^4^Microbiology and Immunology Laboratory of Academic Center of Vitória, Federal University of Pernambuco, Recife, PE, Brazil

## Abstract

*Paullinia cupana* (Guarana) is a native plant of Amazon region that has very traditional importance. Its seeds are rich in bioactive compounds, including tannins, which exhibit relevant properties.* Objective.* This study aimed to evaluate antibacterial, antineoplastic, and immunomodulatory activity of* P. cupana* seeds crude extract (CE) and ethyl-acetate fraction (EAF).* Methods.* Antibacterial activity was evaluated by determination of minimal inhibitory concentration (MIC) and minimal bactericidal concentration (MBC). Antineoplastic activity was evaluated by MTT assays in hepatocellular carcinoma (HepG2), breast adenocarcinoma (MCF-7), ductal carcinoma (T47-D), non-Hodgkin's B cell lymphoma (Toledo), T cell leukemia (Jukart), and Acute Leukemia (HL-60) cell lines. BALB/c mice splenocytes were treated to assess IFN-*γ*, IL-6, IL-17, and IL-10 levels by sandwich ELISA.* Results.* CE and EAF were not toxic to peripheral blood cells and splenocytes. CE and EAF fractions showed a bacteriostatic activity (MIC = 250 *μ*g/mL) and presented IC_50_ values of 70.25 *μ*g/mL and 61.18 *μ*g/mL in HL-60 leukemia cell line. All cytokines evaluated had their levels reduced after treatment, following dose-response model.* Discussion and Conclusion.* Different biological activities were observed for both CE and EAF, suggesting* P. cupana* as a source of bioactive substances, especially tannins that may be used for several diseases treatments.

## 1. Introduction

The use of plants with the aim of treating, preventing, and curing diseases is one of the oldest forms of human medical practice [1]. In this context, many plants used in traditional medicine are being studied, including* Paullinia cupana* Kunth, popularly known as guarana.* P. cupana* is native from the Amazon region, and it is known for its stimulant and medicinal properties and is used for centuries by indigenous communities in the Amazon [2].

Several functional properties have been attributed to guarana and reported in the literature, including antioxidant [3–6] and antifatigue activity [7], reduction of platelet aggregation and thromboxane synthesis* in vitro* [8], and antimicrobial [5, 6] and antidepressant action [9]. Furthermore, guarana also plays an important role in the treatment of cardiovascular diseases, promoting a reduction in blood pressure levels and LDL fraction in an elderly population residing in the Amazon region [10].

These effects observed in* P. cupana* extracts are attributed to secondary metabolites present in its composition. Its seeds contain high amounts of methylxanthine, such as caffeine, theobromine, and theophylline. Some saponins, polyphenols, and especially the tannins are found in extract fractions of guarana seeds [11, 12].

A study by Marcon et al. was able to associate guarana with reduced serum levels of IL-1*β*, IL-6, and IFN-*γ* showing its anti-inflammatory potential. Guarana has also presented effectiveness in the treatment of fatigue associated with chemotherapy in patients with solid tumors [13]. Thus, this article aims to conduct a joint evaluation of antibacterial, antineoplastic, and immunomodulatory activity of the crude extract (CE) and the ethyl-acetate fraction (EAF) of* P*.* cupana*.

## 2. Methods

### 2.1. Plant Obtaining

*Paullinia cupana* Kunth seeds were acquired from Mr. José Augusto de Souza at Alta Floresta region, Mato Grosso, Brazil. All plant materials were identified and processed at Laboratory of Pharmaceutical Biology, Palafito, Pharmacy Department of Maringa State University. A dried specimen was deposited in the Herbarium of the Department of Biology of the State University of Maringa (HUEM#9.065). This material was identified by Professor Dr. Cássia Mônica Sakuragui. Crude extract (CE) and ethyl-acetate fraction (EAF) of* P. cupana* seeds were obtained already lyophilized from Palafito and these materials were prepared by Klein et al. (2012). Quickly, the CE was prepared by Ultra-Turrax (UTC115KT) using acetone : water (7 : 3, v/v) for 20 min. The organic phase was eliminated by rotary evaporator under reduced pressure and lyophilized. The CE was partitioned with ethyl acetate and removed the organic solvent to yield the EAF. The EAF was submitted at solid-phase extraction (SPE). EAF (2.00 mg) was diluted in methanol 20% (1 mL) and passed through SPE cartridge and the volume completed to 25 mL of 20% methanol. 10 *μ*L was used by HPLC according to Klein et al. (2012).

### 2.2. Antibacterial Evaluation

#### 2.2.1. Bacterial Strains

The methicillin-resistant* Staphylococcus aureus* (MRSA) and* Staphylococcus epidermidis* clinical strains were obtained at the University Hospital (Hospital das Clínicas of Federal University of Pernambuco) and preserved at the Laboratory of Microbiology and Immunology (LMB) of the Academic Center of Vitória of Federal University of Pernambuco (CAV-UFPE). These strains were previously identified as MRSA and* Staphylococcus epidermidis* according to* Clinical and Laboratory Standard Institute* guidelines [14]. Reference strains were obtained from* American Type Culture Collection* (ATCC), such as MRSA ATCC 33591, methicillin-sensitive* Staphylococcus aureus* ATCC 29213,* Escherichia coli* ATCC 25922,* Klebsiella pneumoniae* ATCC 29665, and* Pseudomonas aeruginosa* ATCC 27853.

#### 2.2.2. Antibacterial Activity

The antibacterial activity of* P. cupana* extracts and fraction was assessed by the broth microdilution method according to* Clinical and Laboratory Standards Institute* guidelines [14]. Initially, 96-well microlitres plates were filled with Mueller-Hinton broth (MHB), and then each extract was dissolved in 0.5% DMSO and serial diluted to obtain concentrations ranging from 0.5 to 250 *μ*g/mL. Subsequently, the bacterial suspensions were adjusted to 0.5 McFarland standard turbidity and diluted to achieve a final concentration of 10^5^ CFU/mL in each well. The microplates were then incubated at 35 ± 2°C for 24 h. Antimicrobial activity was detected by adding 20 *μ*L of 0.5% triphenyl tetrazolium chloride aqueous solution (TTC, Vetec). The minimal inhibitory concentration (MIC) was defined as the lowest concentration of extracts that inhibited visible growth as indicated by the TCC staining (dead cells are not stained by TTC) [15]. The minimal bactericidal concentration (MBC) was determined by subculturing samples from the wells with concentrations above the MIC on new plates of Mueller-Hinton agar (MHA). The MBC was considered as the lowest concentration of the extracts associated with no bacterial growth. DMSO (0.5%) was used at the same concentration of the experiment to evaluate any possible effects of solvents on bacterial growth. All the experiments were performed in triplicate.

### 2.3. Antineoplastic Evaluation

#### 2.3.1. Toxicity Assay in Peripheral Blood Cells

The assay was performed as previously described by [16]. Peripheral blood mononuclear cells (PBMC) were obtained from heparinized blood from healthy, nonsmoking donors who had not taken any drugs for at least 15 days and had not been drinking alcohol at least 3 days prior to sample collection. Cells were isolated via a standard method of density-gradient centrifugation over Ficoll-Hypaque solution (GE Healthcare). Cells were counted in a Neubauer chamber, and viability was determined by the trypan blue exclusion method. Cells were used only when the viability was 98%. All donors gave informed consent, and the study was approved by the Human Research Ethics Committee of UFPE in the Health Sciences Center (opinion number 1.285.288). Cells were plated in 96-well plates (5.5 × 10^5^ cells/well). After 24 h, the compounds were added (10, 50, 100, and 200 *μ*g/mL), and the cells were incubated for 48 h and then subjected to the MTT assay. After 48 h incubation, 20 *μ*L of MTT was added in each well (0.5 mg/mL). After 3 h, the formazan product of MTT reduction was dissolved in 20% sodium dodecyl sulfate, and the absorbance of the solution at 570 nm was measured with a multiplate reader (EL808, Biotek, USA). The effect of each compound tested was quantified in terms of the absorbance as a percentage of the absorbance of the reduced dye in the DMSO treated group.

#### 2.3.2. Human Tumor Cell Lines and MTT Assay

Cytotoxicity of CE and EAF was tested against six human tumor cell lines: HepG2 (hepatocellular carcinoma), MCF-7 (human breast adenocarcinoma), T47-D (ductal carcinoma), Toledo (non-Hodgkin's B cell lymphoma), Jukart (T cell leukemia), and HL-60* (Acute Leukemia)*. All cell lines were obtained from Rio de Janeiro Tissue Cell Bank. Cells were cultured in RPMI-1640 medium supplemented with 10% fetal bovine serum, 2 Mm glutamine, 100 mg/mL streptomycin, and 100 U/mL penicillin at 37°C with 5% CO_2_.

For the cytotoxicity assay, cells were plated in 96-well plates (10^4^ cells/well). After 24 h, a dimethyl sulfoxide solution of tested compounds (10–200 *μ*g/mL) was added to each well and incubated for 72 h. Control groups were treated with the same amount of dimethyl sulfoxide (0.1%). Inhibition of cell growth was evaluated by MTT method as previously described. IC_50_ values were determined with the viability of four tested doses and the selectivity index (SI) was calculated through the ratio between PBMC IC_50_ and Tumoral Cell IC_50_ values. Extracts were considered as promisors when values of SI were more than 3 [17].

### 2.4. Immunomodulatory Activity

#### 2.4.1. Obtaining Splenocytes

Experimental tests used BALB/c mice (*n* = 9, 45 days old). Animals were provided by Keizo Asami Immunopathology Laboratory (LIKA) (Federal University of Pernambuco, Recife, Brazil). Mice were sacrificed according to the Ethics Committee Guidelines for the Use of Experimental Animals of the Federal University of Pernambuco (opinion number 23076.041556/2015-62).

After sacrifice, mice had their spleens removed and transferred to Petri dishes containing RPMI1640 medium (Gibco) to be crushed in order to obtain splenocytes. Splenocytes suspension was recovered, filtered through Cell Strainer 40 *μ*m (BD Bioscience), and centrifuged at 300 G, 10 min, 6 acceleration, brake 4. If necessary, cell pellet was treated with RBC lysis solution 1x Buffer (eBioscience) to lyse the blood cells present. Splenocytes were counted in a Neubauer chamber and cell viability was determined by trypan blue method. Cytotoxicity was measured by MTT assay. EAF and CE at 10–200 *μ*g/mL doses were added in triplicate.

#### 2.4.2. Splenocytes Culture

Splenocytes were cultured in 24-well plates (2 × 10^6^ per well) in RPMI-1640 medium (Gibco) supplemented with 10% fetal bovine serum (Gibco), 10 mM HEPES (Gibco), and penicillin and streptomycin 200 U/mL (Gibco). Cells were stimulated with ConA at 100 ng/mL. As controls, untreated cells and cells treated with methylprednisolone (100 *μ*M) were utilized. EAF and CE were added to different wells at a concentration of 5, 10, 50, and 100 *μ*g/mL. After incubation with extract, fraction, and controls, plates were incubated at 37°C and 5% CO_2_.

### 2.5. Cytokine Determination

After 48 h of incubation, 1 mL supernatant was collected from each well and used for measurement of cytokines by ELISA kits for mouse following the manufacture's information. Lower limits of detection of ELISA kits were 7.81 pg/mL for IL-6 (BD Biosciences), 15.6 pg/mL for IL-10 (eBiosciences) and IFN-*γ* (BD Biosciences), and 3.90 pg/mL for IL-17A.

### 2.6. Statistical Analysis

OringPro8 was used in IC_50_ calculation. Normal variables distribution was evaluated by Kolmogorov-Smirnov test. Statistical analysis was performed using GraphPad PRISM® software version 6. All experiments were performed in triplicate. Statistical test used in the analysis of cytokines in the culture supernatant was Wilcoxon and the results were considered significant when *p* < 0,05. Variables did not follow normal distribution and were represented in the graphs as median, maximum, and minimum.

## 3. Results

### 3.1. Cytotoxicity Assay

Before performing any biological activity of guarana CE and EAF, cytotoxicity assays in PBMC and splenocytes BALB/c mice were initially assessed. After 48 h, both EAF and CE were not toxic at 200 *μ*g/mL dose in PBMC, demonstrating that both compounds did not show cytotoxicity in front of cells from healthy donors at tested doses. However, at 200 *μ*g/mL both EAF and CE were toxic to splenocytes and cell culture was assessed until 100 *μ*g/mL (5, 10, 50, and 100 *μ*g/mL).

### 3.2. Antineoplastic Assay

**Figure 1:**
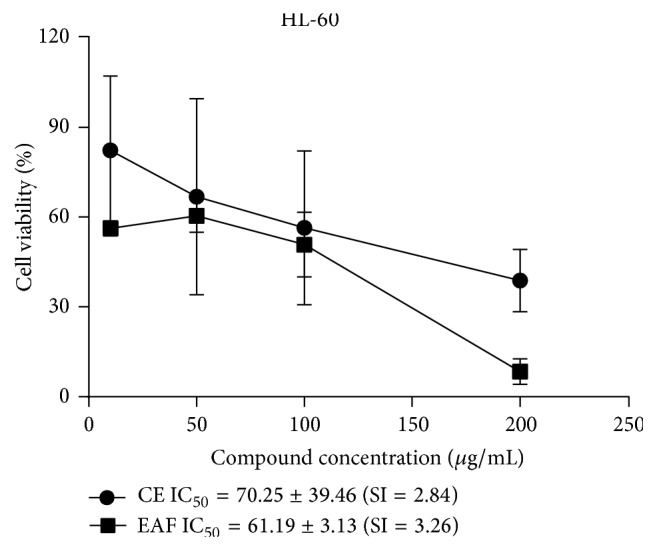
Cell viability, related IC_50_, and selectivity index (SI) of* Paullinia cupana* CE and EAF in HL-60 cells.

In our study was observed antiproliferative activity of guarana CE and EAF in HL-60 cells (70.25 ± 39.46 and 61.18 ± 3.13 *μ*g/mL, resp.) (Figure 1). However, the same was not observed for other cell lines even in the highest tested dose of 200 *μ*g/mL.

### 3.3. Antibacterial Assay

After examination of cytotoxicity, the antibacterial activity was evaluated by the microdilution method, aiming to determine MIC and MBD values. The results showed that EAF and CE showed bacteriostatic activity (MIC = 250 *μ*g/mL); however they do not show bactericidal activity (MBC > 250) (Table 1).

### 3.4. Evaluation of Cytokines Modulation

**Figure 2:**
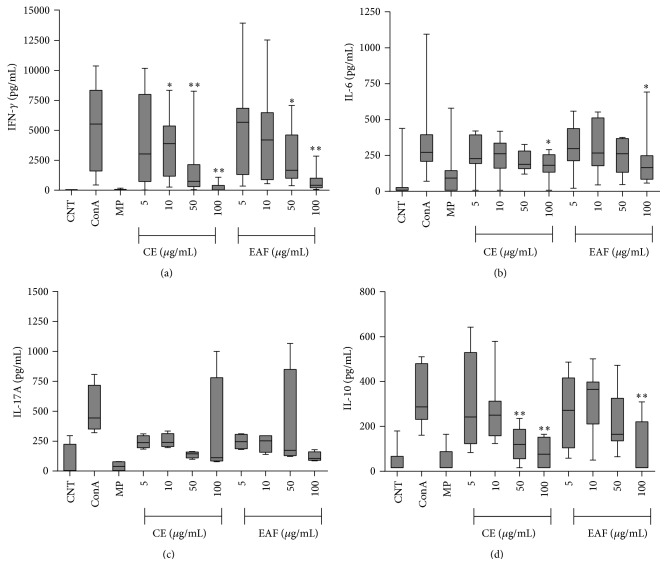
Cytokines measured in splenocytes culture supernatant treated with both crude extract and acetate fraction of* Paullinia cupana* at concentrations of 5, 10, 50, and 100 *μ*g/mL. In the chart there is the comparison of CE and EAF action with controls (CNT = control untreated and ConA = Concanavalin A). The degree of significance is given quantity asterisk (*∗*) and bars represent median, minimum, and maximum.

Since EAF and CE did not show cytotoxicity in splenocytes BALB/c mice at the concentration of 200 *μ*g/mL (data not shown), splenocytes were incubated with extracts to assess the levels of pro- and anti-inflammatory cytokines. Comparisons of cytokines levels in the supernatant are observed at Figure 2.

For IFN-*γ* (Figure 2(a)) both fractions had dose-response behavior: after treatment with the higher dose of the extract, cytokine secretion was lower. There was a greater inhibition by crude extract for all doses tested, except at 5 *μ*g/mL.

IL-6 cytokine (Figure 2(b)) levels were reduced mainly in CE when compared to control (ConA), following dose response again. However, only in 100 *μ*g/mL dose a significant reduction in the secretion of the cytokine for both CE (*p* = 0.0391) and EAF (*p* = 0.0156) was observed.

Both CE and EAF reduced IL-17A levels (Figure 2(c)) in culture supernatant, but it was not statistically significant. Differently, IL-10 (Figure 2(d)) levels showed a significant reduction in 50 and 100 *μ*g/mL doses of CE when compared to ConA control (*p* = 0.0078 and *p* = 0.0039, resp.) and for the dose of 100 *μ*g/mL in EAF (*p* = 0.0078).

## 4. Discussion

**Figure 3:**
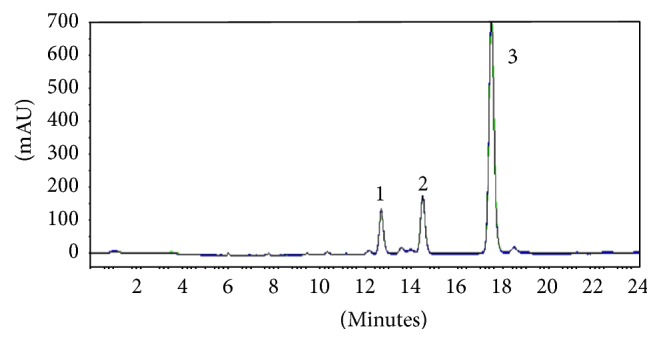
Chromatogram of ethyl-acetate fraction of* Paullinia cupana*, Lot 1. (1) catechin; (2) epicatechin; (3) caffeine.

Medicinal plants are important for pharmacological research, treatment, and prevention of diseases not only when used directly, but also as raw materials for the synthesis of pharmacologically active compounds [1, 18]. In this context, guarana plays an important role as one of the most promising products of flora. Currently popular culture advocates the use of guarana in the form of syrups, employing it in a wide variety of effects such as antidyspeptic; antidiarrheal; physical and mental fatigue, antihemorrhagic; aphrodisiac; nervous system stimulant; cardiovascular tonic; diuretic, among others [19]. Given these numerous properties related to guarana, this article presented together three of its main biological properties through the evaluation of guarana CE and EAF activities. In a previous study EAF was analyzed by high performance liquid chromatography (HPLC) and the presence of methylxanthines and tannins was observed at this guarana fraction [12]. The HPLC-method was revalidated and statistically evaluated, and the concentration of catechin, epicatechin, and caffeine was determined, respectively, *μ*g/mL (*μ*g/mg of EAF): 13.19 (164.88); 16.03 (200.34); 20.84 (260.52) (Figure 3). The results were according to Klein et al. (2012).

Some bioactive compound presenting in plants extracts represents an alternative to new drug discovery [5]. The bacterial resistance, the appearance of new pathogenic microorganisms, and the reappearance of old pathogens represent key points to development of new alternatives drugs [20]. Tannins are some of these compounds that exhibited an antibacterial potential* in vitro* against* Streptococcus mutans* and* S. sobrinus* [21, 22]. In our study, a bacteriostatic effect in methicillin-resistant* Staphylococcus aureus* (MRSA) isolates was observed, considered as an important pathogen responsible for high hospital death rates and economic spending [23]. Since MRSA often present resistant to almost all beta-lactam antibiotics, cephalosporin, and a wide range of other antibiotics, the identification of this property of guarana is seen interesting [24, 25]. Basile and contributors [26] observed better bactericidal effect of acetonic and ethanolic fraction of* P. cupana* in nine Gram positive and negative bacteria suggesting that different extracts types can exert antibacterial activity. Their results can be associated to presence of different compounds found in acetonic and ethanolic fractions that were not found at our analyzed extracts. Distinct antimicrobial activities observed by the same plant may be explained by the presence of a greater amount of bioactive compounds resulting from the extraction method, fraction type, and microorganism utilized. Thus, different effects found in studies using* P. cupana* fractions can be attributed to differences in fractions, extraction methods, and the quantity of tannins, phenolic compounds, and caffeine in each fraction [6].

Other beneficial effects related to guarana consist in the antineoplastic and chemoprotective activity that can be associated to tannins present in its composition. Such effects were demonstrated by Fukumasu and contributors in many studies* in vivo* using* P. cupana* [27–29]. Some studies describe the relation of tannins with antineoplastic activity in human tumor cells [30–33]. In this work we found an antineoplastic activity of FAE and CE against HL-60 with statistically significant effect. Thus, tannins can probably influence their effect. Tannins were found in other plant species and presented antineoplastic activity against HL-60 [34]. Differently, guarana antineoplastic property was not observed for other cell lines even in the highest dose tested. Difference at results with previous studies can be explained by variation of the plant used and the type of study. Recently, a study conducted by Hertz and colleagues evaluated the antiproliferative effect of guarana in breast tumor cell line (MCF-7) as well as its association with chemotherapeutic agents. However, they did not observe a decrease in cell proliferation at guarana without chemotherapeutic association [19]. According to Hertz et al. (2015) we do not observe antineoplastic activities of guarana extract and fraction at this breast cancer lineage.

Effects of guarana and its extracts in the immune system are few reported. Recently, a study using an animal model verified that guarana seeds present an immunomodulatory activity with expression decrease of NF-K*β* and COX2, as well as significant reduction of cytokines expression like IL-1, IL-6, and TNF-*α* [35]. Results showed here demonstrated that guarana decreases levels of inflammatory cytokines IFN-*γ*, IL-6, and IL-10, following a dose-response pattern. CE and EFA decreased IL-17A levels, but the results were not significant. Immunomodulatory action of guarana is probably associated with catechins and caffeine present in our extracts and fractions composition. Caffeine acts as an antagonist of adenosine receptors, inhibiting the enzyme adenosine monophosphate phosphodiesterase and increasing the amount of intracellular cAMP (cyclic adenosine monophosphate) that can reduce the production of proinflammatory cytokines such as IFN-*γ* [36–38]. In a second study, an* in vitro* protocol was used to evaluate the effect of guarana in inflammatory markers levels. PBMCs were treated with and without guarana (5 g/mL), glucose (15 mM), and insulin (1 mU/mL). They also evaluated* in vivo* plasma cytokine levels in healthy individuals supplemented with guarana (90 mg/day) for 14 days. In both* in vitro* and* in vivo* tests a decrease in the levels of proinflammatory cytokines IL-1*β*, IL-6, TNF, and IFN-*γ* [39] was observed, confirming our findings. Tannins such as procyanidins have been studied to present an anti-inflammatory effect by inhibition of proinflammatory cytokines like IL-6 and TNF-*α* [40]. EAF also presents procyanidins in its composition [12] and these compounds can be associated with its immunomodulatory profile observed at this study.

## 5. Conclusion

*Paullinia cupana* extracts have potential to be studied, mainly because of their low cytotoxic potential in normal cells. Both CE and EAF of guarana have an antimicrobial and immunomodulatory potential of being an important source of new therapeutic approaches. Simultaneous presence of different bioactivities observed in this study* in vitro* for both CE and EAF suggests that* Paullinia cupana* is an interesting source of bioactive substances, especially tannins that may be used in the future for the treatment of several diseases.

## Figures and Tables

**Table 1:** Minimal inhibitory concentration (MIC) and minimal bactericidal concentration (MBC) of *Paullinia cupana* CE and EAF. Values are shown at *µ*g/mL.

	CE		EAF	
	MIC	MBC	MIC	MBC
	*µ*g/mL		*µ*g/mL	
*Escherichia coli *ATCC 25922	250	>250	250	>250
Clinically isolated of *Staphylococcus epidermidis*	250	>250	250	>250
*Staphylococcus aureus* ATCC 29213	250	>250	250	>250
Methicillin-resistant *Staphylococcus aureus* (MRSA) ATCC 33591	250	>250	250	>250
LMB 02	250	>250	250	>250
LMB 03	250	>250	250	>250
LMB 04	250	>250	250	>250
LMB 05	250	>250	250	>250
LMB 06	250	>250	250	>250
LMB 07	250	>250	250	>250
LMB 08	250	>250	250	>250
LMB 09	250	>250	250	>250
LMB 10	250	>250	250	>250
